# Lung Cancer Cells Infiltration into a Mandibular Follicular Cyst

**DOI:** 10.1155/2023/7297821

**Published:** 2023-07-17

**Authors:** Stefano Marelli, Martina Ghizzoni, Matteo Pellegrini, Andrea Scribante, Gioacchino D'Ambrosio, Domenico Sfondrini

**Affiliations:** ^1^Maxillo-Facial Surgery Unit, Fondazione IRCCS Policlinico San Matteo, Pavia 27100, Italy; ^2^Section of Dentistry, Department of Clinical, Surgical, Diagnostic and Pediatric Sciences, University of Pavia, Pavia 27100, Italy; ^3^Maxillofacial Surgery and Dental Unit, Fondazione IRCCS Cà Granda Ospedale Maggiore Policlinico, Milan 20122, Italy; ^4^Department of Biomedical, Surgical and Dental Sciences, University of Milan, Via della Commenda 10, Milan 20122, Italy; ^5^Department of Molecular Medicine, Anatomic Pathology Unit, University of Pavia and Fondazione IRCCS San Matteo Hospital, Pavia 27100, Italy

## Abstract

**Introduction:**

The oral cavity is a rare site for other organs' tumor metastases. The incidence rate ranges from 1% to 3% of all oral malignancies. Metastases more frequently localize in the mandible, especially in the molar area. Metastases within odontogenic cysts are extremely rare, thus in the literature, only a few cases have been reported. The follicular cyst is one of the most frequent cysts of the jaws. Radiologically it shows as a unilocular lesion with a sclerotic border, characterized by a homogenous radiolucency that incorporates the crown of the unerupted tooth.

**Methods:**

A 76-year-old female patient, affected by stage IV of lung adenocarcinoma, reported pain on the left mandible border, alongside lip dysesthesia. The ortho-panoramic radiograph showed a follicular cyst in the posterior left side of the mandible, with involvement of the ascending branch region. The cyst was surgically removed together with the impacted tooth. A histopathological examination of the specimen confirmed the diagnostic suspect of a follicular cyst, but in the cystic wall, focal infiltration of epithelial neoplastic cells was also found. The immunohistochemical analysis showed the presence of three different markers: CK 7+, TTF1+/−, and P40−. These markers identify the cells as metastatic lung carcinoma.

**Results:**

Secondary tumor spreading in the jaws' area is rare (3% of all malignant lesions). Despite odontogenic cysts can undergo a dysplastic transformation (affecting the epithelial covering in ≤1% of the cases), infrequently these neoplastic cells can be related to secondary tumor spreading in a cystic wall. This report describes a metastatic localization within a mandibular follicular cyst.

**Conclusions:**

Malignant cancers' metastasis in the oral district remains a rare and unexplored condition, especially when metastases are located in odontogenic cysts. In this circumstance, surgical removal and histopathological examination are strongly recommended.

## 1. Introduction

Follicular cyst or dentigerous cyst (DC) is one of the most frequent cysts that can affect the upper and lower jaws, representing 17%, and up to 25% of total maxillary cysts [1, 2].

Table 1 briefly summarizes the most frequent odontogenic cysts and their clinical presentation, diagnostic modalities, and treatment.

Typically, this developmental cyst is associated with impacted mandibular third molars and more rarely with maxillary canines [3].

Based on etiology, odontogenic cysts can be divided into inflammatory or developmental. The first category is composed of periapical cysts, residual cysts, and paradental cysts, whereas the second category is composed of DC, eruption cyst, lateral periodontal cyst, gingival cyst, odontogenic keratocyst, ortho-keratinizing odontogenic cyst, and glandular odontogenic cyst [4].

According to World Health Organization, the follicular cyst is the result of fluid accumulation between the reduced enamel epithelium and the dental follicle of an unerupted tooth [5].

Being mainly asymptomatic, DC can be diagnosed through a radiographic routine examination or as a further investigation related to an unerupted tooth. Some patients may refer to pain and swelling symptoms, usually corresponding to an infectious state of the cyst [6]. In the case of large cysts, patients may also report lip dysesthesia due to nerve compression. More rarely, loss of dental occlusion due to a fracture of the jaws may be noticed. DC often presents a slow growth pattern and a predisposition to centrifugal expansion against the jaw's bone cortex [7].

Through ortho-panoramic radiograph and cone-beam computed tomography (CBCT) investigations, most DCs appear as clearly defined unilocular lesions with a sclerotic border, characterized by a homogenous radiolucency. The cyst incorporates the crown of the unerupted tooth, originating from its cementoenamel junction. The transversal growth pattern typically progresses toward a single plate, buccal or lingual; a bi-cortical expansion is uncommon. Among the complications that can occur, the most common ones are cyst infection, and if undiagnosed for a long time, cortical plates' resorption, which can lead to mandibular or maxillary fractures [8–10].

Even though odontogenic cysts are benign lesions, in some cases they can undergo neoplastic degeneration into primary intraosseous carcinomas or uncommon jaws cancers, arising from odontogenic epithelial residues [11, 12]. These neoplastic lesions are asymptomatic in most cases, leading to a delay and hindrance in the diagnosis of oral cancer [13]. The specific mechanisms responsible for the malignant transformation of the benign cystic lining of odontogenic cysts are still unknown. However, the most supported hypothesis is the chronic inflammation trigger that can or cannot be associated with a genetic predisposition [11].

A radiographic classification divides follicular cysts into three types: a central variety, in which the cyst envelopes the tooth crown, a lateral variety, in which the cyst is localized along the tooth root beside a partial crown involvement, and a circumferential variety, in which the tooth is completely enclosed in the cyst, from the crown to the root [3].

The oral cavity is a rare site for other organs' tumor metastases the incidence rate range goes from 1% to 3% maximum of all oral malignancies [14].

Metastases can localize both in soft and hard tissues, being the mandible the most frequent site, specifically the molar area. The primary provenance of the original tumor is breast, lung, and kidney [15–17]. Metastatic neoplasms affecting the jaws can be localized peri-orally and intra-orally. They can involve both soft and hard tissues. Mandible, and specifically the molar area, is more affected if compared with the maxilla. As concerns soft tissues, adherent gingiva is the most represented site for cancer metastasis, followed by the tongue [16, 18].

Since the diagnosis is quite challenging, the clinician's recognition of the lesion and the pathologist's determination of the original site of the primary tumor must be combined, approaching a multidisciplinary one [19].

Cancer cells circulating in the bloodstream or lymphatic vessels, survive and settle in secondary organs, by extravasation through vessels' walls, becoming then proper cancer metastases through proliferation [20].

However, the real pathogenesis of metastatic malignancies in the jaws remains partially unknown [14].

Very rarely metastatic cells can localize inside the odontogenic cyst's wall.

This case report aims to describe a rare lung cancer cell infiltration into a mandibular follicular cyst. Only four cases of malignancies metastases in odontogenic cysts of maxillary bones have been documented in the literature before.

## 2. Case Report

### 2.1. Diagnosis and Etiology

A 76-year-old female patient T.E. was referred to the Unit of Maxillo-Facial Surgery of IRCCS Policlinico San Matteo, Pavia, Italy. She reported pain on the left mandible border, alongside lip dysesthesia.

The patient affected by stage IV of lung adenocarcinoma is currently in treatment with osimertinib for primary lung cancer and denosumab for bone metastases.

By analyzing her medical history, it emerges that CT scan with a contrast medium was previously performed. It showed a solid lesion at the level of the right lung apex with a diameter of 35 mm × 26 mm, surrounded by a ground-glass perimeter. In addition, in both lungs there was evidence of numerous sub-centric nodular images, extending to all lobes. This finding appears attributable to bilateral neoplastic milia. Pathologic lymph nodes were also evident in Barety's lodge, aortopulmonary area, and sub-carenal site, as well as bilateral ileal.

Biopsy confirmed a cytologic picture consistent with lung adenocarcinoma (TTF 1+), PD-L1 <1%, mutated EGFR, BRAF, and KRAS wild type, ALK, and ROS-1 negative.

Magnetic resonance imaging spine with and without contrast medium, showed findings compatible with secondarisms involving vertebral bodies of C4, C7, D3, D7, D11, L1, L4, and S1.

**Figure 1:**
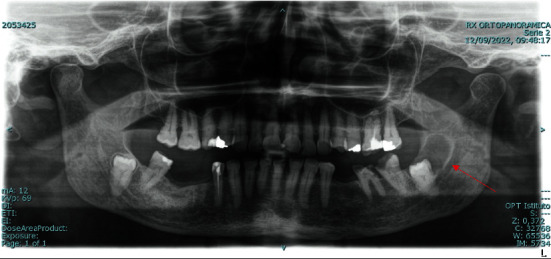
Panoramic radiograph: a follicular cyst was identified at element 3.8 (red arrow).

Because of the symptoms, a panoramic radiograph was performed, which showed an impacted wisdom tooth with a follicular cyst on the left side of the mandible, with a mild involvement of the ascending branch region (Figure 1).

**Figure 2:**
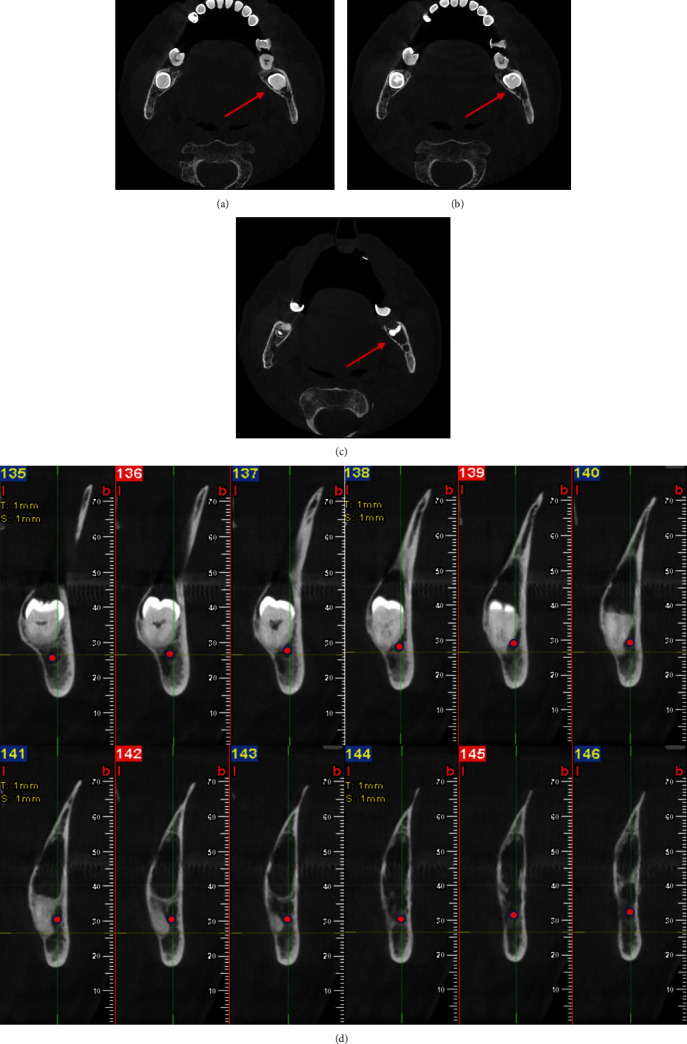
(a–c) 3D dimensions and boundaries of the follicular cyst (red arrow through CBCT axial section and (d) relationship with inferior alveolar nerve (IAN), located buccally (redpoint) through cross-sectional imaging.

A CBCT was prescribed to determine the real extension of the lesion, its anatomical boundaries, and relationships with the inferior alveolar nerve through axial sections (Figures 2(a), 2(b), and 2(c)) and cross-sectional sections (Figure 2(d)). Subsequently, the treatment plan was decided and analyzed with the patient.

### 2.2. Treatment Objectives

The treatment involves the surgical removal of the follicular cyst (cystectomy) and the subsequent extraction of the lower third molar (3.8) in osteomucosal impaction, with direct wound closure. Care was taken to avoid any injury to the inferior alveolar nerve, which is located buccally to the tooth roots.

### 2.3. Treatment Alternatives

Depending on the cyst dimension, localization, and anatomical relationships with adjacent structures, DC treatment can vary from enucleation (cystectomy) followed by tooth removal to marsupialization. In case of wide cyst dimensions with an important cortical bone erosion (mandible or maxilla), it is possible to perform the marsupialization to avoid jaw fractures [22, 23]. This procedure consists of a cyst incision, and subsequent suture of cystic edges to the adjacent epithelial tissue to create a pouch. This communication between the oral cavity and cyst, reducing the intra-cystic pressure, allows cystic volume reduction, through bone ossification. Both clinical and radiological follow-ups need to be performed [24]. In this specific case, no marsupialization occurred since the cyst's dimension permits its complete removal without any risk of bone fractures or compromising adjacent anatomical structures.

### 2.4. Treatment Progress

After local anesthetic infiltration with a vasoconstrictor (mepivacaine + adrenaline 1:100,000, Optocain, Molteni Dental S.r.l, Milan, Italy), a crestal incision through retromandibular trigone extended mesially to the fornix and distally to the mandibular branch were performed with no. 15 blade (Pic solution, PikDare S.p.A, Como, Italy), and a mucoperiosteal flap was raised. Cortical erosion in the retromandibular trigone showed the presence of the follicular cyst. Then, osteotomy was carried out, with a 2.3 mm TPX round burr (Stryker, Kalamazoo, MI, USA), alongside the surgical extraction of the element 3.8. The remaining follicular cyst was then removed and sent to the Unit of Pathological Anatomy of IRCCS Policlinico San Matteo, Pavia.

After cyst enucleation, a curettage of the surgical site associated with saline solution cleansing was conducted. Finally, interrupted resorbable stitch sutures (4.0 Vicryl Rapide, Ethicon, Somerville, NJ, USA) were performed throughout the interested region.

### 2.5. Treatment Results

After the cystectomy, the surgical wound healed without any complications. At the 1-month follow-up, symptoms disappeared. Histopathological examination of the surgical specimen confirmed the diagnostic suspect of a follicular cyst.

Macroscopic findings showed a grayish flap with a dimension of 1 cm referring to the cystic wall in toto. Microscopically, edema, blood extravasation, accumulation of macrophages, and minimal chronic flogosis were observed.

**Figure 3:**
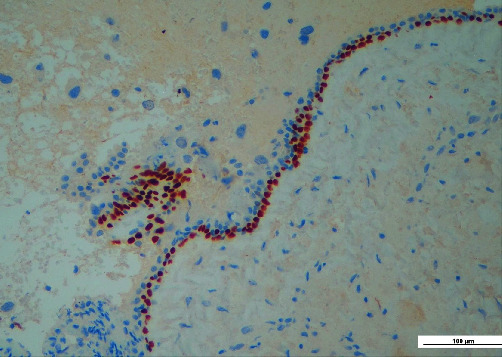
Thin layer of squamous epithelium covering the cyst wall.

**Figure 4:**
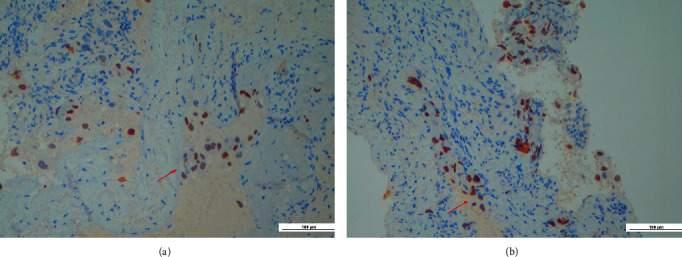
(a and b) The neoplastic cells show a nuclear reaction with TTF1 (Red arrow).

**Figure 5:**
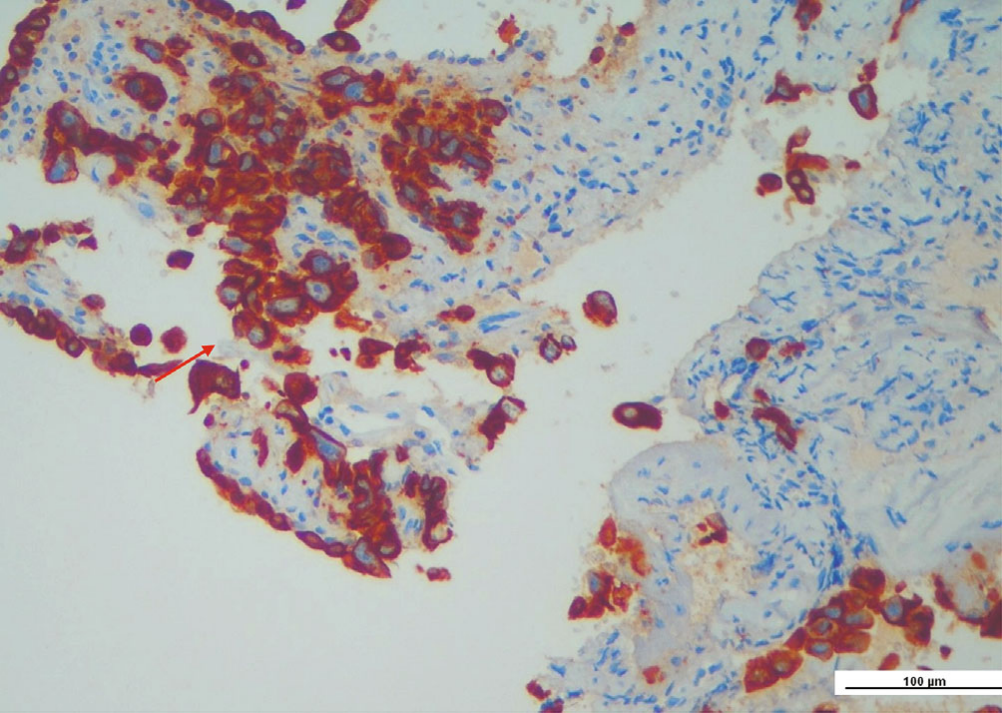
The neoplastic cells are strongly immunoreactive for CK7 (red arrow).

**Figure 6:**
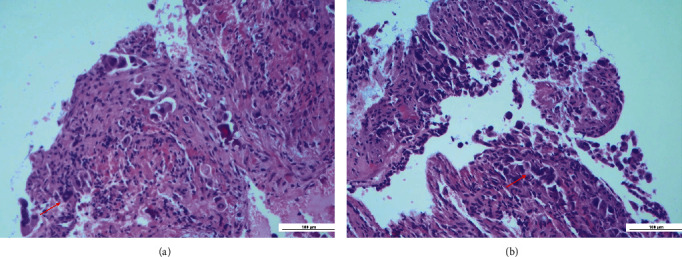
(a and b) Infiltration of neoplastic cells in the thickness of the cyst wall (EE 40×, red arrow).

Histopathological examination revealed an odontogenic cyst wall lined by a thin squamous epithelium expressing p40 (Figure 3); in the wall's thickness, it was also found focal infiltration of neoplastic cells (Figures 4(a) and 4(b)), immunoreactive for CK7 (Figure 5), and TTF1 (Figures 6(a) and 6(b)), consistent with lung adenocarcinoma.

Although histological examination confirmed the diagnostic suspect, the lesion was only treated with cyst enucleation since the patient was already following an oncologic treatment with osimertinib and denosumab.

## 3. Discussion

We aim to describe a rare case of metastasis of a primary tumor discovered inside the wall of an odontogenic follicular cyst in a patient with lung adenocarcinoma. The features that make this case report notable are the histopathological and immunohistochemical analysis findings.

During odontogenesis, specialized epithelial cells and odontogenic ecto-mesenchymal cells interact, leading to tooth formation. Thus, a considerable range of neoformations can involve the jawbone, ranging from benign lesions to malignant ones [25, 26].

Secondary tumor spreading in the jaws' area is rare, representing up to 3% of all malignant lesions in this region [27]. Radiologically, jaws' metastases often are described as radiopaque lesions with unclear borders and cortical involvement, unlike cystic lesions [28].

Females seem to be more likable to be affected since breast cancer is the prevalent malignancy to cause metastases to the maxillary bones. However, other tumors can spread to jawbones, such as the lung, kidney, adrenal gland, bone, and colon [29].

In literature, several cases of oral manifestations of primary cancer have been described.

Although bone appears to be the most common site for metastases of solid tumors (breast, prostate, thyroid gland, kidney, and lung), which metastases can rarely be classified as bone-only [30–32].

In the oral cavity, metastases can affect hard and soft tissues, and jawbones are twice affected as gingiva [33].

The pathogenesis still nowadays remains unclear. Cancer cells can disseminate through lymphatic or hematic streams. Bones with active bone marrow (vertebrae, pelvis, ribs, skull, and femur) are elected areas for tumor cell storage [34].

Active bone marrow appears to be an inviting nest to initiate metastatic processes for different reasons: sinusoidal blood spaces are an easy barricade to access, bone marrow is also rich in growth factors that can help tumor cells' proliferation and invasion, and finally, there is a directly proportional relationship between metastasis and hematopoiesis, indeed metastatic process enhances hematopoiesis [34–37].

Adults' lower jaw has a significant marrow content, which could explain the specific localization of metastases in the mandible area, especially in the angles and in the ascending ramus (molar area) [36].

Unconventional sites, such as the mandibular condyle or maxillary gingiva, can be colonized by the secondary spreading of primary cancer that can manifest with subtle or even without any symptoms [38–40].

Radiological findings related to mandibular metastases are a-specific, thus biopsy and subsequent histopathological analysis are needed to confirm any previous diagnostic suspect. Moreover, if the patient already has been diagnosed with cancer, the pathologist should determine as well if there is a correspondence between the primary cancer and oral findings through histomorphological and immunohistochemical analysis. It is important to highlight that generally, oral metastasis suggests an advanced stage of cancer [29, 41].

Odontogenic cysts can experience dysplastic transformation, affecting the epithelial covering. Tumors typically originating from the epithelial covering of odontogenic cysts are benign odontogenic neoplasms like odontoma or Pindborg tumor [42, 43].

Rarely, the cystic epithelium can undergo malignant transformation, becoming mucoepidermoid carcinoma or squamous cell carcinoma [44, 45].

Clinically and radiologically, these malignant neoplasms show comparable characteristics to benign odontogenic neoplasms or non-transformed original cysts of the jaws. Their typical localization seems to be the mandibular third molar area.

Neoplastic transformation of odontogenic cysts into carcinomatous, mucoepidermoid, or even squamous, happens in very low percentages of cases (≤1%), and most of them (80%) are found in the lower jaw [46].

Long-lasting chronic inflammation could be a risk factor leading to a malignant transformation of the epithelial lining [47].

On the other hand, malignancies metastases in odontogenic cysts of maxillary bones appear to be an extremely rare condition, with very few cases in the literature. They seem to be often related to female patients, with primary breast cancer [48].

Currently, in the literature, only four cases have been described.

Malignant melanoma has been found in a cyst wall of the anterior maxilla [49]. Three breast carcinomas were discovered in a cyst located in the posterior mandible. The diagnosis was in three out of the four cases of a periapical cyst, and only in one case of a DC [48, 50, 51].

Thus, the present case is the second to report a DC with focal infiltration of cancer cells, and the first one to describe specifically lung carcinoma cells colonization, inside its wall.

The frequent association with a periapical cyst can be explained by the great capillary network formed by chronic inflammation that can capture circulating malignant cells. Moreover, newly formed vessels, in the inflamed tissue are not sealed tightly, making them more accessible for cancer cells. However, little to nothing is known about the localization of neoplastic cells, inside DC's wall [52, 53].

Assuming that inflammatory tissue attracts neoplastic cells, we should expect the same incidence of metastases inside odontogenic cysts in both maxillary bones. Since this is not the case, probably there are further mechanisms for why lower jaws are more frequently involved, that to this day remains unknown and needs further in-depth studies.

This study presents some limitations. This is a single case report, with only a 3-month follow-up. Moreover, not every time a cyst is removed an immunohistochemical analysis is performed, making these findings difficult to identify. Finally, only a few cases have been described in the literature, since it is an extremely rare condition. Future studies need to be conducted to evaluate metastatic cell infiltration in odontogenic cysts to better understand their physio-pathological and spreading mechanisms.

## 4. Conclusion

Malignant cancer metastasis in the oral district remains a rare and unexplored condition, especially in odontogenic cysts. This case report describes a lung cancer cell infiltration into a mandibular follicular cyst. The uncommon localization of metastatic cells is the distinctive characteristic of this report. Suspicious lesions like these in the oral cavity should be further investigated when associated with malignancy's medical history since often oral metastasis can be exchanged for benign conditions.

## Figures and Tables

**Table 1:** Most frequent odontogenic cysts [21].

Type of cyst	Clinical presentation	Diagnostic modalities	Differential diagnosis	Management
Radicular cyst	It is caused by trauma or dental caries leading to dental pulp necrosis Apical epithelial cells (rests of Malassez) are triggered by pulpal inflammation, forming a Periapical cyst (PC)	Pulp testing, radiological, and histopathologic evaluation	Periapical granuloma, lateral radicular cysts	Root canal treatment, apicectomy, and tooth extraction or cyst removal
DC	It is caused by the accumulation of fluids between follicular epithelium and the crown of an unerupted tooth	Radiological and histopathologic evaluation	Odontogenic keratocyst, unicystic ameloblastoma, ameloblastic fibroma, adenomatoid odontogenic tumor	Enucleation or marsupialization. In selected cases, the impacted tooth remains in situ
Odontogenic keratocyst	Typically localized in the posterior mandible, it is considered to be an odontogenic cyst that originates from dental lamina's residues	Radiological and histopathologic evaluation	DC, ameloblastoma	Surgical excision followed by osseous curettage and eventually chemical fixation with Carnoy's solution application

## Data Availability

The authors confirm that the data supporting the findings of this study are available within the article.

## Supplementary Materials

Supplementary materials CARE checklist for case reports.
