# Supplementary material for: Lung Cancer Cells Infiltration into a Mandibular Follicular Cyst
Source: Case Rep Dent. 2023 Jul 17;2023:7297821. doi: 10.1155/2023/7297821 (PMC10365922; doi:10.1155/2023/7297821)
Supplement: Supplementary materials — CARE checklist for case reports. [file 7297821.f1.docx]

**Supplementary file:** Care checklist.


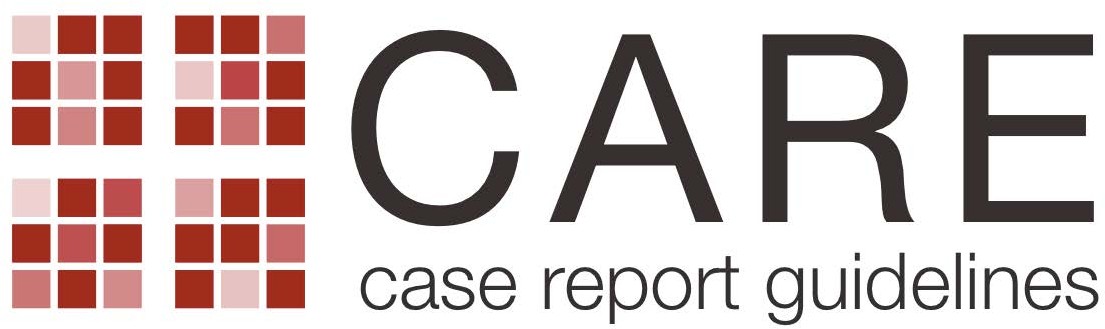
CARE Checklist of information to include when writing a case report
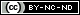


**Topic Item Checklist item description Reported on Line**

**Title 1** The diagnosis or intervention of primary focus followed by the words “case report” 1

**Key Words 2** 2 to 5 key words that identify diagnoses or interventions in this case report, including "case report" 50

**Abstract**

**(no references)**

**3a** Introduction: What is unique about this case and what does it add to the scientific literature? 39

**3b** Main symptoms and/or important clinical findings 36

**3c** The main diagnoses, therapeutic interventions, and outcomes 37

**3d** Conclusion—What is the main “take-away” lesson(s) from this case? 47

**Introduction 4** One or two paragraphs summarizing why this case is unique (**may include** reference**s**) 81 and 94

**Patient Information 5a** De-identified patient specific information 99

**5b** Primary concerns and symptoms of the patient 102

**5c** Medical, family, and psycho-social history including relevant genetic information 100

**5d** Relevant past interventions with outcomes 100

**Clinical Findings**

**Timeline**

**Diagnostic Assessment**

**Therapeutic Intervention**

**Follow-up and Outcomes**

1. Describe significant physical examination (PE) and important clinical findings 103
2. Historical and current information from this episode of care organized as a timeline N.R.

**8a** Diagnostic testing (such as PE, laboratory testing, imaging, surveys). 105

**8b** Diagnostic challenges (such as access to testing, financial, or cultural) N.R.

**8c** Diagnosis (including other diagnoses considered) 105 and 147

**8d** Prognosis (such as staging in oncology) where applicable N.R.

**9a** Types of therapeutic intervention (such as pharmacologic, surgical, preventive, self-care) 132

**9b** Administration of therapeutic intervention (such as dosage, strength, duration) 132

**9c** Changes in therapeutic intervention (with rationale) N.R.

**10a** Clinician and patient-assessed outcomes (if available) 144

**10b** Important follow-up diagnostic and other test results 150

**10c** Intervention adherence and tolerability (How was this assessed?) N.R.

**10d** Adverse and unanticipated events N.R.

**Discussion 11a** A scientific discussion of the strengths AND limitations associated with this case report 215 and 233

**11b** Discussion of the relevant medical literature **with references** 169

**11c** The scientific rationale for any conclusions (including assessment of possible causes) 224

**11d** The primary “take-away” lessons of this case report (without references) in a one paragraph conclusion 240

**Patient Perspective 12** The patient should share their perspective in one to two paragraphs on the treatment(s) they received N.R.

**Informed Consent 13** Did the patient give informed consent? Please provide if requested . . . . . . . . . . . . . . . . . . . . . . . . . . . . . . . . . . . . . . **Yes No**
